# Supplementary material for: The Provenance of Slovenian Milk Using 87Sr/86Sr Isotope Ratios
Source: Foods. 2021 Jul 27;10(8):1729. doi: 10.3390/foods10081729 (PMC8393216; doi:10.3390/foods10081729)
Supplement: Supplementary file 1 [file foods-10-01729-s001.zip › foods-1287017-supplementary.pdf]

Supplementary Materials

The provenance of Slovenian milk using $^{87}Sr/^{86}Sr$ isotope ratios

Staša Hamzić Gregorčič[1,2], Nives Ogrinc[1,2], Russell Frew[3], Marijan Nečemer[2], Janja Vrzel[4], Lidija Strojnik[1,2] and Tea Zuliani[1,2,*]

[1] Department of Environmental Sciences, Jožef Stefan Institute, Jamova 39, Ljubljana, Slovenia; stasa.gregorcic@ijs.si (S.H.G.); nives.ogrinc@ijs.si (N.O.); lidija.strojnik@ijs.si (L.S.); tea.zuliani@ijs.si (T.Z.)
[2] Jožef Stefan International Postgraduate School, Jamova 39, Ljubljana, Slovenia; S.H.G.; N.O.; marijan.necemer@ijs.si (M.N.); L.S.; T.Z.
[3] Department of Chemistry, University of Otago, PO Box 56, Dunedin, New Zealand
[4] JR-AquaConSol GmbH, Graz, Austria
[*] Correspondence: tea.zuliani@ijs.si; Tel.: +386 1 477 3542

This supplement material describes the optimisation and validation of an analytical method for $^{87}Sr/^{86}Sr$ isotope ratio determination in milk. The protocol includes: reagents and standard solutions, sample pre-treatment, decomposition of the sample matrix, Sr-matrix separation and method validation.

1. Reagents and standard solutions

Hydrochloric acid (30% (w/w) HCl, Suprapur) and hydrogen peroxide (30% (w/w) H_2O_2) were obtained from Merck Ltd. (Darmstadt, Germany). Nitric acid (68-70% (w/w) HNO_3, Superpure) was obtained from Carlo Erba Reagents Srl (Milan, Italy). For elemental screening, the standard solutions containing Sr and Rb were prepared by dilution of the single-element ICP standards (1000 mg/L, Certipur, Merck, Darmstadt, Germany). Rhodium (Rh), as an internal standard, was prepared by dilution of the single-element ICP standard (1000 mg/L, Certipur, Merck, Darmstadt, Germany). SPS-SW1 Surface-water-trace elements, was obtained from Spectrapure Standards (Oslo, Norway). Ultrapure water with a resistivity of 18.2 MΩ cm was obtained from a Milli-Q Element System (Merck Millipore, Watertown, MA, USA) and used throughout this work for the preparation of standard solutions and diluting concentrated acids and samples. For separation of Sr from the sample matrix, Sr-selective resin (100-150 µm, TrisKem International, Bruz, France) was used. For the quality control of the pre-treatment techniques, namely the total Sr and Rb concentrations, CRMs NIST RM 8435 (whole milk powder; National Institute of Standards and Technology, Gaithersburg, USA) with declared fat content 21.3% and IAEA-153 (milk powder; International Atomic Energy Agency, Vienna Austria) were used. The fat content of IAEA-153 is not declared. This reference material was analysed for interlaboratory comparison to validate the optimised method and determine its $^{87}Sr/^{86}Sr$ isotope ratio value.

2. Sample pre-treatment

As no matrix certified reference material (CRM) for Sr isotope analysis exists, the preliminary experiments were carried out using a sample of whole milk powder labelled PM (whole milk powder, 26% fat content, Pomurske Mlekarne, Murska Sobota) that was purchased on the local market in Slovenia.

Before the sample pre-treatment step, all glass and plastic wares were soaked in 10% (v/v) analytical grade HNO_3 overnight, rinsed with Milli-Q water and dried in the oven. Samples were pretreated using microwave-assisted acid digestion system UltraWAVE (Single Reaction Chamber Microwave Digestion System; Milestone, Soristone, Italy). Freeze-dried and homogenised milk sample (0.30 g) was weighed into Teflon (PTFE) tube, and 6 ml of concentrated HNO_3 was added. The tubes

were closed and subjected to microwave digestion. Samples were pretreated using the program, as shown in Table S1. Further, the resulting solutions were quantitatively transferred into 30 ml polyethylene (PE) graduated vials and filled up to 15 ml with Milli-Q water. Samples were stored at 4 °C until further processing. After each mineralisation cycle, a cleaning cycle was performed with 6 ml of diluted HNO_3 (1:1, v/v) to eliminate cross-contamination.

Table S1. Microwave-assisted acid digestion program used for pre-treatment of milk sample.

Step	Time	Ramp °C/min	T final	Hold time	Power
1	00:20:00	11°C/min	240°C	30 min	1500 W

After mineralisation, sample solutions were evaporated to near dryness on a sand bath at T ≤ 90°C (IKA-C-MAG HP 10, IKA-Werke GmbH & Co., Staufen im Breisgau, Germany). Residual sample materials were redissolved in concentrated HNO_3 and H_2O_2 to destroy any residual organic matter. If organic matter was still present, the evaporation of sample was repeated until the complete destruction of the organic matter. Samples were then dissolved in 8M HNO_3 for subsequent Sr/matrix separation. The Sr selective resin (0.30 g) was weighed directly into 2 ml columns. The columns were rinsed with Milli-Q water, activated with 6M HCl and preconditioned with 8M HNO_3. Measurements of Sr and Rb concentrations in mineralised milk samples and the Sr fraction were performed by ICP-MS (7900x, Agilent Technologies Inc., Tokyo, Japan) to follow the mass balance. For the quality control of the measurements, SPS-SW1 was analysed at the beginning and end of each analytical sequence. Good agreement between certified and determine Rb and Sr concentrations were obtained.

3. Decomposition of the sample matrix

The method was optimised in terms of completeness of mineralisation, chemical recovery of Sr isolated from the sample matrix, minimal contamination, and turnaround time. The blanks of analyte-free media were prepared using the same materials and reagents as for the samples. To monitor the percentage recovery of target analytes and the effectiveness of matrix destruction, total concentrations of Sr were measured by ICP-MS after sample pre-treatment and after the Sr separation procedure (Table S2). The non-digested residual organic matter can strongly affect Sr/Rb separation efficiency on the column. Thus, Sr recovery after Sr/matrix separation step gives us information about the effectiveness of the matrix destruction. The experiments were carried out on a sample of whole milk powder (PM – Pomurske mlekarne), which was previously analysed, and its Sr concentration was estimated to be 2.90 ± 0.08 mg/kg.

The method was optimised based on the recovery of Sr after the Sr/matrix separation using varying sample size (0.30 and 0.70 g) and acid digestion volumes (4 and 6 ml) and acid mixtures (HNO_3 and/or a mixture of HNO_3 and H_2O_2) (Table S2).

Table S2. Comparison of the Sr concentrations obtained after microwave digestion of freeze-dried milk samples (mean ± standard deviation; n = 3).

Sample weight [g]	Reagents	Sr concentration after pretreatment [mg/kg]	Sr concentration after Sr-matrix separation [mg/kg]	Sr recovery [%]
0.70	4 ml HNO_3	2.88 ± 0.08	2.10 ± 0.78	72.5 ± 7.2
0.70	6 ml HNO_3	2.90 ± 0.10	2.27 ± 0.66	78.5 ± 10.0
0.70	5 ml HNO_3 + 1 ml H_2O_2	2.89 ± 0.08	2.04 ± 0.49	70.7 ± 0.8
0.30	4 ml HNO_3 + 2 ml H_2O_2	2.94 ± 0.13	2.59 ± 0.23	88.6 ± 6.7
0.30	6 ml HNO_3	2.98 ± 0.07	2.91 ± 0.28	97.9 ± 1.6

Given the results of the microwave method, all tested samples and reagents quantities gave similar Sr concentrations. Therefore, it can be concluded that total Sr recovery from the sample was obtained. As for accurate isotope determination, 100% recovery is desirable to avoid possible isotope fractionation, and the method with the highest recovery was selected. Low Sr recoveries after the separation (~75%) were found for the sample size of 0.70 g, most probably due to the presence of larger quantities of residual organic matter. Based on this observation, the optimal sample size to digest was

found to be 0.30 g with 6 ml HNO_3, which achieved high Sr recovery (~98%) and was used for further analysis.

To evaluate the accuracy and precision of pre-treatment techniques, NIST SRM 8435 and IAEA-153 were analysed for Sr concentration. The results found for the CRMs were compared with their corresponding certified values and are reported in Table S3. At a 95% confidence level, good agreement with the certified values was found in all cases (Student's *t*-test, $p < 0.05$). The relative standard deviation (RSD) for three replicates of each standard ranges from 0.55 to 1.92%. The Sr concentrations in method blanks from three independent analyses were 0.0026 ± 0.0006 mg/kg.

Table S3. Determined Sr concentrations after pre-treatment in certified reference materials, NIST SRM 8435 and IAEA-153 (mean ± standard deviation; n = 3).

CRM	Sample weight [g]	Pretreatment method	Certified Sr [mg/kg]	Determined Sr [mg/kg]	Sr recovery [%]
NIST 8435	0.30	microwave	4.35 ± 0.50	4.12 ± 0.26	94.6 ± 6.0
IAEA-153	0.30	microwave	4.09 ± 0.34 [a]	3.99 ± 0.05	97.3 ± 1.8

[a] Values are informative according to the certificate (based on dry weight).

4. Sr-matrix separation

Regarding the Sr and Rb content in milk samples, optimising the procedure for efficient Sr loading and Rb removal has been performed. Before the loading of the sample onto the Sr selective resin, pretreated samples were evaporated to near dryness and redissolved in 1 ml of 8M HNO_3 to obtain concentrations ranging between 300 – 700 ng Sr/ml before being loaded onto the column and 30 – 70 ng Sr/ml after the column, which is the optimal concentration range for Sr isotope ratio determination by MC-ICP-MS.

Strontium is strongly retained on the Sr resin with the increasing HNO_3 concentration of the sample and eluting solution [25]. While 3M HNO_3 has been shown to adequately separate Sr from Rb during chromatographic extraction separation from biological, environmental, and nuclear waste samples [47], 8M HNO_3 was shown to maximise the separation of other matrix elements such as Ba and Ca [48,49]. As milk contains high concentrations of these elements, 8M HNO_3 was therefore selected. The samples were loaded on the resin in 8M HNO_3, and the resin was rinsed with 8M HNO_3 to maximise the elution of Rb and other possible interferences. Given the high Rb/Sr ratio in milk samples, it was difficult to remove all Rb in a single separation step, as is the case with biological tissues [50] and geological materials [51]. Therefore, after the first separation, the solution was evaporated again to near dryness and redissolved in 1 ml of 8M HNO_3 and submitted for the second separation on a freshly prepared column. The Rb removal was monitored by measuring its concentration before and after the chromatographic extraction procedure by ICP-MS. For this experiment, five different freeze-dried milk samples with different Rb/Sr ratios were analysed. The results are presented in Table S4.

Table S4. The efficiency of Rb removal.

Sample	Before separation Rb [ng/mL]	Rb/Sr ratio	After 1st separation Rb [ng/mL]	After 2nd separation Rb [ng/mL]
Milk 1	2216	8.1	0.103	< 0.005
Milk 2	1297	4.1	0.042	< 0.005
Milk 3	8402	28.0	0.278	< 0.005
Milk 4	5455	17.9	0.115	< 0.005
Milk 5	4858	16.1	0.257	< 0.005

As far as the efficiency of the Sr elution from the resin is concerned, Sr can be eluted from the column with either Milli-Q water or diluted HNO_3. In the present study, Milli-Q water [12,14,20] and

0.05M HNO3 [11,52,53] were used to determine the appropriate eluent. Better Sr yield was achieved by using Milli-Q water.

With a smaller resin size (0.20 g), it is possible that a small amount of strontium was flushed away from the column, which resulted in lower recovery compared to 0.30 g resin. The lowest extraction efficiency was observed using 0.70 g resin, probably due to the inadequate quantity of mobile phase. The optimal size of the resin was found to be 0.30 g and was used in further experiments (Figure S1).

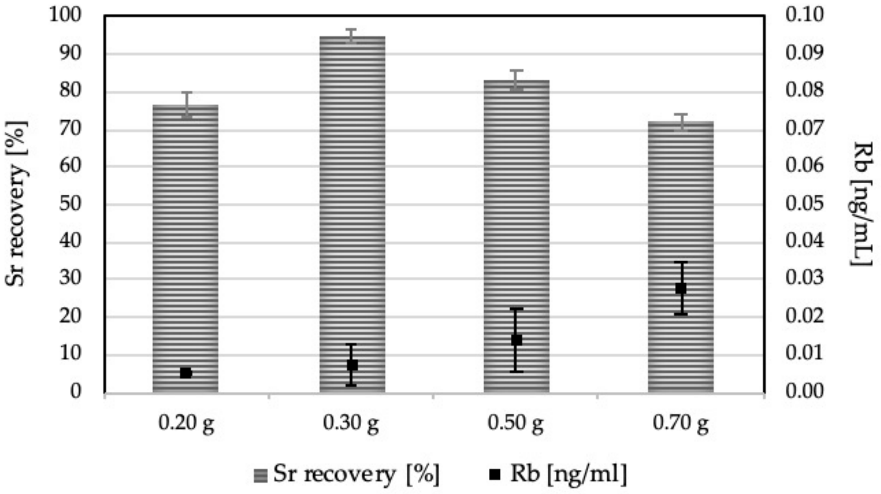

Figure S1. Extraction efficiency using different amounts of the Sr-Spec resin.

5. Method validation

Unfortunately, no matrix-matched certified reference materials are available for the $^{87}Sr/^{86}Sr$ isotope ratio determination in milk. Consequently, the optimised method's repeatability, precision, and trueness were tested by analysis of the candidate reference material, IAEA-153.

Eight independent replicates were prepared in two days (4 each day). One sample from each day was measured three times. All measurements were carried out under optimised experimental parameters. Estimation of the measurement uncertainty for the Sr isotope ratio was done according to the guide to the expression of measurement uncertainty JCGM 100:28 and ISO GUM [54,55], applying the uncertainty propagation law without using second-order covariances. Combined uncertainty included contributions from the ion intensity measurements, mass bias corrections, and contributions from certified values of relevant ratios. The results are presented in Table S5 and were found to be comparable.

Table S5. $^{87}Sr/^{86}Sr$ isotope ratios[1] for IAEA-153 milk sample.

Laboratory	$^{87}Sr/^{86}Sr$	Average	SD
JSI	0.70831		
	0.70838		
	0.70819		
	0.70825	0.70829	0.00008
	0.70835		
	0.70823		
	0.70828		
	0.70824	0.70828	0.00005
	0.70829		
	0.70824		

	0.70830	0.70828	0.00003
	0.70835		
	0.70824		
	0.70830	0.70829	0.00005
JSI value +/- U (k=2)		*0.70828*	*0.00015*
**UO**	0.708288		
	0.708294		
	0.708286	0.708289	0.000004

[1] Strontium isotope ratio determinations were carried at JSI out using a Nu II multi-collector ICP-MS instrument (Nu Instruments, Ametek Inc., UK) fitted to an Aridus IITM Desolvating Nebulizer System (Teledyne Cetac, Omaha, Nebraska, USA). At UO, determinations were conducted using a Nu Plasma HR MC-ICP-MS (Nu Instruments, Ametek Inc., UK) operating in low mass resolution mode coupled with a DSN-100 (Ametek, USA) desolvating sample introduction system.

The measurement uncertainty was larger than the standard deviations since the NIST 987 is the main contributor. The trueness was tested by comparing the $^{87}Sr/^{86}Sr$ isotope ratio independently obtained from the Department of Chemistry at the University of Otago in New Zealand (UO). UO prepared and measured three independently prepared samples. Based on the results, it can be concluded that the optimised method described above is fit-for-purpose for the determination of the $^{87}Sr/^{86}Sr$ isotope ratios of milk.

Table S6. The whole dataset of authentic milk sample analysis including the description of location, season and year of production, geological background, stable isotope ratio of oxygen ($^{18}O/^{16}O$) in milk water and $^{13}C/^{12}C$, $^{15}N/^{14}N$ and $^{34}S/^{32}S$ in casein, elemental analysis in the freeze dry samples determined with XRF.

Basic data											XRF										
Location ID	Location	Latitude	Longitude	Season	Year	Geology	$\delta^{18}O_w$ ‰	$\delta^{13}C_{cas}$ ‰	$\delta^{15}N_{cas}$ ‰	$\delta^{34}S_{cas}$ ‰	Ca (mg/g)	K (mg/g)	Cl (mg/g)	S (mg/g)	P (mg/g)	Zn (mg/g)	Br (mg/g)	Rb (mg/g)	Sr (mg/g)	Ca/Sr	K/Rb
C1	Sežana	45.70862	13.87428	summer	2014	Cretaceous: Carbonate Rocks and Flysch	-4.5	-26.5	3.6	1.5	7.62	10.7	8.59	2.23	9.25	24.6	29.9	10.4	2.23	3.42	1.03
C1	Sežana	45.70862	13.87428	winter	2014	Cretaceous: Carbonate Rocks and Flysch	-6.9	-26.0	3.8	1.0	8.80	11.9	8.91	2.60	8.05	27.4	27.8	8.48	2.07	4.25	1.40
C2	Kozina	45.60928	13.93719	summer	2014	Cretaceous: Carbonate Rocks and Flysch	-2.1	-26.8	5.8	6.4	8.93	10.4	5.98	2.54	8.84	31.0	26.3	19.6	1.40	6.38	0.53
C2	Kozina	45.60928	13.93719	winter	2014	Cretaceous: Carbonate Rocks and Flysch	-4.4	-23.6	4.9	4.9	9.21	11.4	7.12	2.54	7.86	30.3	24.2	10.5	2.77	3.32	1.09
C2	Kozina	45.60928	13.93719	winter	2015	Cretaceous: Carbonate Rocks and Flysch	-4.5	-18.9	5.6	5.2	n.d.	n.d.	n.d.	n.d.	n.d.	n.d.	n.d.	n.d.	n.d.	n.d.	n.d.
C3	Brkini	45.55083	14.06222	summer	2014	Cretaceous: Carbonate Rocks and Flysch	-5.2	-23.7	4.6	5.1	6.86	8.96	5.42	2.04	9.25	28.2	19.6	14.4	1.46	4.70	0.62
C3	Brkini	45.55083	14.06222	winter	2014	Cretaceous: Carbonate Rocks and Flysch	-7.0	-26.1	5.2	5.9	8.80	12.5	6.90	2.45	8.18	22.5	24.6	28.3	0.94	9.38	0.44
C3	Brkini	45.55083	14.06222	winter	2015	Cretaceous: Carbonate Rocks and Flysch	-6.1	-22.2	5.5	7.2	n.d.	n.d.	n.d.	n.d.	n.d.	n.d.	n.d.	n.d.	n.d.	n.d.	n.d.
C4	Postojna	45.77504	14.21382	summer	2014	Cretaceous: Carbonate Rocks and Flysch	-3.9	-25.9	5.1	5.1	n.d.	n.d.	n.d.	n.d.	n.d.	n.d.	n.d.	n.d.	n.d.	n.d.	n.d.
C4	Postojna	45.77504	14.21382	winter	2014	Cretaceous: Carbonate Rocks and Flysch	-6.3	-20.5	5.8	4.3	7.46	8.8	7.07	2.51	8.06	28.1	17.1	25.1	1.12	6.66	0.35
C4	Postojna	45.77504	14.21382	winter	2015	Cretaceous: Carbonate Rocks and Flysch	-6.1	-24.4	5.3	3.6	n.d.	n.d.	n.d.	n.d.	n.d.	n.d.	n.d.	n.d.	n.d.	n.d.	n.d.
C5	Logatec	45.91761	14.23516	summer	2014	Cretaceous: Carbonate Rocks and Flysch	-4.5	-24.9	4.6	4.4	9.40	14.3	9.74	2.78	9.25	36.3	20.3	37.3	1.50	6.27	0.38
C5	Logatec	45.91761	14.23516	winter	2014	Cretaceous: Carbonate Rocks and Flysch	-6.8	-22.7	5.1	5.4	8.20	10.8	6.98	2.50	8.83	31.2	20.0	13.0	2.32	3.53	0.83
C6	Šmihel	45.95101	13.73780	winter	2014	Cretaceous: Carbonate Rocks and Flysch	-6.8	-24.3	5.8	4.4	7.98	9.69	6.71	2.72	8.67	29.9	13.2	13.2	2.40	3.33	0.73
J1	Bohinj	46.30065	13.94305	summer	2014	Jurassic: Carbonate Rocks	n.d.	-24.1	3.8	5.0	7.79	10.2	7.42	2.24	7.95	27.5	13.9	27.0	1.39	5.60	0.38
J1	Bohinj	46.30065	13.94305	winter	2014	Jurassic: Carbonate Rocks	-7.1	-24.3	3.9	4.5	7.55	10.4	7.26	2.23	7.04	28.4	15.2	17.1	0.95	7.94	0.61
J2	Žužemberk	45.83072	14.92945	summer	2014	Jurassic: Carbonate Rocks	-5.0	-22.8	5.2	5.9	8.04	9.99	6.50	2.21	7.17	26.1	18.1	17.2	1.21	6.64	0.58
J2	Žužemberk	45.83072	14.92945	winter	2014	Jurassic: Carbonate Rocks	-6.9	n.d.	n.d.	n.d.	8.83	11.1	7.41	2.74	8.41	32.4	16.8	16.7	1.32	6.69	0.66
J2	Žužemberk	45.83072	14.92945	winter	2015	Jurassic: Carbonate Rocks	-7.8	-21.3	5.7	4.1	n.d.	n.d.	n.d.	n.d.	n.d.	n.d.	n.d.	n.d.	n.d.	n.d.	n.d.
J3	Podpeč	45.97324	14.41981	summer	2014	Jurassic: Carbonate Rocks	-5.4	-20.2	5.1	5.3	8.22	9.53	6.19	2.47	7.63	32.1	12.7	12.5	3.03	2.71	0.76
J3	Podpeč	45.97324	14.41981	winter	2014	Jurassic: Carbonate Rocks	-6.4	-19.6	5.2	5.4	9.79	11.5	7.06	2.93	9.41	37.7	12.0	10.0	1.56	6.28	1.15
J4	Kamenje	45.85574	15.15377	summer	2014	Jurassic: Carbonate Rocks	-5.5	-21.1	6.0	4.8	7.38	10.7	7.13	2.35	6.57	29.4	17.2	16.7	1.23	6.00	0.64
J4	Kamenje	45.85574	15.15377	winter	2014	Jurassic: Carbonate Rocks	-7.2	-19.5	5.4	4.9	7.85	10.5	6.88	2.49	8.09	30.2	24.2	16.0	2.15	3.65	0.66
J5	Vinica	45.46142	15.25357	summer	2014	Jurassic: Carbonate Rocks	-5.4	-22.6	5.5	6.7	7.36	10.6	7.22	2.30	7.72	28.7	23.4	18.1	1.24	5.94	0.59
J5	Vinica	45.46142	15.25357	winter	2014	Jurassic: Carbonate Rocks	-6.9	-20.0	5.4	4.7	8.14	9.70	5.80	2.51	8.72	34.0	9.73	12.7	3.26	2.50	0.76
J6	Šmarje pri Jelšah	45.97639	14.61882	winter	2014	Jurassic: Carbonate Rocks	-7.2	-19.2	5.9	3.3	8.49	11.2	7.64	2.72	8.09	31.1	10.3	12.8	3.06	2.77	0.88
N1	Šentilj	46.68544	15.70966	summer	2014	Neogene: Carbonate Rocks	-5.5	-24.0	6.6	4.8	7.84	9.45	6.39	2.56	8.00	24.6	11.7	14.4	2.96	2.65	0.66
N1	Šentilj	46.68544	15.70966	winter	2014	Neogene: Carbonate Rocks	-7.1	-20.1	6.1	3.9	8.71	10.6	7.32	2.80	8.50	31.9	10.7	17.1	3.23	2.70	0.62
N2	Kamnik	46.22219	14.60712	summer	2014	Neogene: Deposits	-5.3	-22.4	5.8	4.2	6.93	9.79	6.43	2.05	6.44	21.3	12.6	10.9	1.90	3.65	0.90
N2	Kamnik	46.22219	14.60712	winter	2014	Neogene: Deposits	-6.6	-20.9	5.4	4.5	7.94	9.79	5.85	2.10	6.75	24.6	8.99	10.0	0.89	8.93	0.98
N3	Ponikva	46.25371	15.44393	summer	2014	Neogene: Deposits	-5.4	-22.9	6.0	3.3	7.63	9.99	6.63	2.31	7.69	28.3	9.43	16.0	2.57	2.97	0.62
N3	Ponikva	46.25371	15.44393	winter	2014	Neogene: Deposits	-7.0	-19.8	6.2	3.5	8.78	11.5	7.83	3.10	9.55	32.6	10.6	13.1	2.47	3.55	0.88
N4	Rogaška Slatina	46.23378	15.63860	summer	2014	Neogene: Deposits	-5.3	-22.7	5.9	2.9	8.34	11.2	7.17	2.55	8.24	33.6	9.93	14.9	2.50	3.34	0.75
N4	Rogaška Slatina	46.23378	15.63860	winter	2014	Neogene: Deposits	-7.5	-24.4	6.0	4.7	8.76	11.2	7.69	3.08	8.72	30.4	9.92	12.6	2.99	2.93	0.89
T1	Maribor	46.55463	15.64563	winter	2014	Triassic: Carbonate Rocks	-7.2	-19.7	6.0	1.3	8.11	10.9	7.34	2.62	8.05	32.4	11.0	11.5	3.24	2.50	0.95
T2	Tolmin	46.18696	13.75652	summer	2014	Triassic: Carbonate Rocks	-3.2	-25.7	3.4	4.7	10.1	12.9	9.05	2.76	7.30	27.9	15.7	20.9	1.65	6.12	0.62
T2	Tolmin	46.18696	13.75652	winter	2014	Triassic: Carbonate Rocks	-6.4	-24.1	4.2	5.1	7.64	10.1	7.22	2.66	8.34	30.3	17.9	17.1	1.80	4.24	0.59
T3	Planina	46.04773	14.21534	winter	2014	Triassic: Carbonate Rocks	-7.3	-20.5	5.7	3.5	7.31	9.83	6.82	2.15	7.20	31.0	19.7	14.1	1.36	5.38	0.70
T3	Planina	46.04773	14.21534	winter	2015	Triassic: Carbonate Rocks	-8.7	-20.4	6.1	2.4	n.d.	n.d.	n.d.	n.d.	n.d.	n.d.	n.d.	n.d.	n.d.	n.d.	n.d.
T4	Velike Lašče	45.83366	14.63623	summer	2014	Triassic: Carbonate Rocks	-5.2	-24.0	5.1	5.1	9.12	11.5	7.25	2.39	8.53	29.1	22.0	11.9	2.60	3.51	0.97
T4	Velike Lašče	45.83366	14.63623	winter	2014	Triassic: Carbonate Rocks	-7.2	-22.6	4.8	5.5	8.50	9.80	6.32	2.54	8.24	30.1	19.3	13.2	1.49	5.70	0.74
T5	Vrhe	46.18810	15.01356	summer	2014	Triassic: Carbonate Rocks	-5.6	-25.4	5.1	3.7	9.01	11.6	7.68	2.37	7.80	30.6	12.6	15.6	4.75	1.90	0.74
T5	Vrhe	46.18810	15.01356	winter	2014	Triassic: Carbonate Rocks	-7.3	-22.5	5.6	2.3	8.63	11.6	7.82	2.66	9.72	27.5	11.8	17.1	3.54	2.44	0.68
P1	ibnica na Pohorj	46.53564	15.26751	summer	2014	Paleogene: Deposits	-5.1	-22.4	6.1	4.9	9.59	13.2	8.57	2.80	8.84	34.8	14.5	18.4	2.61	3.67	0.72
P1	ibnica na Pohorj	46.53564	15.26751	winter	2014	Paleogene: Deposits	-7.2	-20.3	6.2	4.7	8.10	10.8	7.25	2.54	7.78	29.6	9.27	16.7	1.63	4.97	0.65
P2	Vuzenica	46.59800	15.16536	summer	2014	Paleogene: Deposits	-5.3	-23.5	6.6	7.0	8.48	11.7	7.66	2.31	7.47	31.4	11.3	17.1	2.83	3.00	0.68
P2	Vuzenica	46.59800	15.16536	winter	2014	Paleogene: Deposits	-7.2	-21.0	6.1	5.3	9.00	11.3	7.99	3.04	9.04	30.2	9.44	14.7	2.59	3.47	0.77
P3	Vitanje	46.38151	15.29503	summer	2014	Paleogene: Deposits	-5.0	-23.4	5.8	4.0	8.51	10.6	6.88	2.36	7.80	28.7	11.3	17.4	2.16	3.94	0.61
P3	Vitanje	46.38151	15.29503	winter	2014	Paleogene: Deposits	-7.0	-21.0	6.3	3.1	9.11	11.1	8.06	3.27	9.66	28.5	13.6	17.1	2.02	4.51	0.65
P4	Slovenj Gradec	46.50779	15.07791	summer	2014	Paleogene: Deposits	-5.7	-22.7	5.7	4.4	7.91	11.6	7.29	2.48	7.86	27.2	13.0	35.5	2.23	3.55	0.33
P4	Slovenj Gradec	46.50779	15.07791	winter	2014	Paleogene: Deposits	-7.2	-21.7	5.8	4.7	8.03	10.4	7.01	2.66	7.95	27.2	10.9	17.1	2.00	4.02	0.61
P5	Mozirje	46.33926	14.95994	summer	2014	Paleogene: Deposits	-4.2	-25.5	6.7	6.5	7.80	10.2	6.54	2.14	9.25	36.3	14.5	10.1	4.18	1.87	1.01
P5	Mozirje	46.33926	14.95994	winter	2014	Paleogene: Deposits	-6.6	-21.1	5.6	3.9	8.64	10.8	7.64	2.83	7.74	34.1	12.9	17.1	2.15	4.02	0.63
P6	Zavodnje	46.42414	15.01712	winter	2014	Paleogene: Igneous Rocks	-7.1	-21.0	6.1	4.6	8.93	11.1	7.76	3.25	9.62	31.2	12.3	13.0	3.57	2.50	0.85
Q1	Savinjska dolina	46.30849	14.89704	summer	2014	Quaternary: Deposits	-5.4	n.d.	n.d.	n.d.	8.44	11.2	7.44	2.73	9.20	32.4	17.4	14.6	2.02	4.18	0.77
Q1	Savinjska dolina	46.30849	14.89704	summer	2014	Quaternary: Deposits	-5.7	-23.3	5.6	2.9	7.75	10.7	7.51	2.25	7.09	29.4	15.4	14.5	1.97	3.93	0.74
Q1	Savinjska dolina	46.30849	14.89704	winter	2014	Quaternary: Deposits	-7.3	-20.7	5.6	4.1	8.37	10.7	7.69	2.84	8.42	26.4	14.7	17.1	1.79	4.68	0.63
Q2	Prevalje	46.54691	14.91991	summer	2014	Quaternary: Deposits	-5.3	-24.6	5.8	3.9	n.d.	n.d.	n.d.	n.d.	n.d.	n.d.	n.d.	n.d.	n.d.	n.d.	n.d.
Q2	Prevalje	46.54691	14.91991	winter	2014	Quaternary: Deposits	-7.4	-21.4	4.5	4.2	8.29	11.2	8.00	2.92	8.31	29.9	11.1	17.1	1.83	4.53	0.65
Q3	Dravograd	46.58922	15.02460	summer	2014	Quaternary: Deposits	-5.2	-24.2	5.9	4.8	8.22	11.7	7.69	2.48	9.12	34.7	12.8	28.1	4.43	1.86	0.42
Q3	Dravograd	46.58922	15.02460	winter	2014	Quaternary: Deposits	-7.3	-21.7	5.8	4.6	9.10	11.6	8.74	3.57	10.2	31.4	11.6	19.2	2.92	3.12	0.60
Q4	Braslovče	46.28804	15.03957	winter	2014	Quaternary: Deposits	-7.1	-20.1	5.7	4.2	8.11	10.3	6.41	2.01	7.62	30.7	12.6	11.7	2.04	3.98	0.88
Q4	Braslovče	46.28804	15.03957	winter	2015	Quaternary: Deposits	-8.8	-19.3	6.1	3.3	n.d.	n.d.	n.d.	n.d.	n.d.	n.d.	n.d.	n.d.	n.d.	n.d.	n.d.
Q5	Škale	46.38925	15.09451	winter	2014	Quaternary: Deposits	-6.9	-20.3	6.0	4.5	8.56	10.8	7.33	2.90	8.37	32.6	14.1	12.3	2.29	3.74	0.88
Q6	Sevnica	46.01295	15.29799	winter	2014	Quaternary: Deposits	-7.0	-20.2	5.1	4.9	8.22	10.3	6.78	2.43	7.74	27.9	13.1	11.1	1.95	4.22	0.93
Q7	lovenske Konjice	46.33665	15.42204	summer	2014	Quaternary: Deposits	-5.4	-21.5	5.7	4.9	8.42	10.0	6.42	2.53	7.16	28.1	10.6	22.1	1.30	6.48	0.45
Q7	lovenske Konjice	46.33665	15.42204	winter	2014	Quaternary: Deposits	-7.0	-19.2	6.1	4.6	10.4	13.5	8.65	3.58	11.3	35.9	14.5	22.5	2.68	3.88	0.60
Q8	lovenska Bistrica	46.39199	15.57278	winter	2014	Quaternary: Deposits	-7.4	n.d.	n.d.	n.d.	8.44	10.6	7.81	3.20	9.09	25.9	13.1	11.7	2.78	3.04	0.91
Q9	Brežice	45.90793	15.59578	summer	2014	Quaternary: Deposits	-5.2	-22.3	5.1	3.6	8.96	11.9	7.57	2.52	9.36	33.7	17.1	20.7	2.46	3.64	0.57
Q9	Brežice	45.90793	15.59578	winter	2014	Quaternary: Deposits	-7.4	-19.5	5.4	3.8	8.93	11.4	7.65	2.90	8.21	31.8	13.6	12.2	2.54	3.52	0.93
Q10	Ptuj 1	46.42006	15.86960	summer	2014	Quaternary: Deposits	-5.6	-19.8	5.9	3.8	7.82	10.7	8.51	2.26	7.79	27.3	14.8	13.7	2.63	2.97	0.78
Q10	Ptuj	46.42006	15.86960	winter	2014	Quaternary: Deposits	-7.0	-19.3	5.8	3.9	8.42	11.0	7.17	2.52	7.95	31.2	10.8	13.9	2.48	3.40	0.79
Q10	Ptuj	46.42006	15.86960	winter	2014	Quaternary: Deposits	-6.9	-18.5	6.2	2.8	7.52	9.75	5.81	2.06	6.30	29.1	10.8	11.5	2.10	3.58	0.85
Q10	Ptuj 2	46.42006	15.86960	winter	2014	Quaternary: Deposits	-6.9	-18.4	6.8	4.6	8.37	10.5	6.43	2.53	8.96	34.3	7.95	11.9	2.33	3.59	0.88
Q10	Ptuj	46.42006	15.86960	winter	2015	Quaternary: Deposits	-6.9	-18.6	5.7	3.7	n.d.	n.d.	n.d.	n.d.	n.d.	n.d.	n.d.	n.d.	n.d.	n.d.	n.d.
Q11	Gornja Radgona	46.67660	15.99125	summer	2014	Quaternary: Deposits	-6.0	-20.1	5.7	4.2	8.94	11	7.21	2.71	9.24	36.1	12.5	13.3	3.30	2.71	0.83
Q11	Gornja Radgona	46.67660	15.99125	winter	2014	Quaternary: Deposits	-7.2	-19.1	6.2	4.8	8.38	10.9	6.69	2.50	8.91	32.4	8.54	13.6	3.23	2.59	0.80
Q12	Radenci	46.64402	16.04111	summer	2014	Quaternary: Deposits	-6.3	-20.8	6.1	4.5	8.17	10.4	6.60	2.25	7.29	33.0	11.2	17.1	3.60	2.27	0.61
Q12	Radenci	46.64402	16.04111	winter	2014	Quaternary: Deposits	-6.7	-19.4	6.1	4.6	8.33	10.4	6.13	2.24	7.67	28.4	11.4	9.71	2.49	3.35	1.07
Q13	Murska Sobota	46.65485	16.16190	summer	2014	Quaternary: Deposits	-6.3	-20.6	6.2	5.1	8.76	10.9	7.56	2.48	8.00	32.2	13.8	17.1	3.60	2.43	0.64
Q13	Murska Sobota	46.65485	16.16190	winter	2014	Quaternary: Deposits	-7.0	-19.8	5.7	5.5	9.73	12.1	7.69	2.66	9.79	34.4	10.5	14.7	3.57	2.73	0.82
Q13	Murska Sobota	46.65485	16.16190	winter	2015	Quaternary: Deposits	-6.9	-19.4	7.7	7.2	n.d.	n.d.	n.d.	n.d.	n.d.	n.d.	n.d.	n.d.	n.d.	n.d.	n.d.
Q14	Ljutomer	46.51955	16.19726	summer	2014	Quaternary: Deposits	-6.0	-20.9	6.4	4.6	8.81	11	6.86	2.49	9.21	34.3	12.9	17.1	3.19	2.76	0.64
Q14	Ljutomer	46.51955	16.19726	winter	2014	Quaternary: Deposits	-6.9	-18.9	6.2	5.4	n.d.	n.d.	n.d.	n.d.	n.d.	n.d.	n.d.	n.d.	n.d.	n.d.	n.d.
Q15	Polzela	46.28095	15.07375	winter	2014	Quaternary: Deposits	-6.9	-19.6	5.6	4.1	8.56	11.1	7.52	2.95	9.26	28.4	11.5	9.8	1.57	5.45	1.13
Q16	Korena	46.52806	15.77623	summer	2014	Quaternary: Deposits	-4.8	n.d.	n.d.	n.d.	7.42	10.9	7.27	2.30	7.02	26.9	9.16	21.5	2.69	2.76	0.51
Q16	Korena	46.52806	15.77623	winter	2014	Quaternary: Deposits	-7.2	-21.7	5.2	3.7	8.89	11.0	6.83	2.69	8.55	32.5	9.20	17.1	1.98	4.49	0.64
Q17	Zahodno Goričko	46.80051	16.22926	summer	2014	Quaternary: Deposits	-6.3	-21.5	6.3	4.5	8.37	10.5	6.96	2.19	7.33	30.3	9.04	17.1	2.81	2.98	0.61
Q17	Vzhodno Goričko	46.80051	16.22926	winter	2014	Quaternary: Deposits	-6.8	-19.5	6.7	5.1	7.97	10.0	6.14	2.29	7.83	34.5	9.07	14.3	2.49	3.20	0.70
